
JOURNAL INFORMATION
==============================
NLM Title Abbreviation: Inter Econ
Journal ID: Inter Econ
NLM Journal ID: 101086399

Inter Economics

ISSN: 0020-5346

ARTICLE INFORMATION
==============================
PMCID: PMC7584869
PMID: 33132415
DOI: 10.1007/s10272-020-0925-x
Article ID: 925
Article version: 1
Subjects: Letter from America

A Vote for the Economy? A Vote Against Democracy

Bourguignon Jiffer j.bourguignon@zbw.eu

grid.461649.8 0000 0001 1019 0408 ZBW, Neuer Jungfernstieg 21, 20347 Hamburg, Germany
Electronic publication date: 2020 Oct 24
Print publication date: 2020
Volume: 55
Issue: 5
First page: 339
Last page: 340
Copyright: © The Author(s) 2020
License: Open Access: This article is distributed under the terms of the Creative Commons Attribution 4.0 International License (https://creativecommons.org/licenses/by/4.0/).
Open Access funding provided by ZBW — Leibniz Information Centre for Economics.
License URL: https://creativecommons.org/licenses/by/4.0/

issue-copyright-statement: © The Author(s) 2020

==============================

When the text books are written, 2020 will go down in the annals of American history as exceptional for a number of reasons. This presidential election year has seen the first global pandemic in a hundred years, unemployment rates dip toward Great Depression-era levels, police violence spurred by systemic racism that has incited civic unrest reminiscent of the 1968 race riots and private citizens arming themselves to the teeth in order to take their security into their own hands amidst fears of "defunding the police." It has endured wildfires burning up and down the entire west coast of the US that have killed dozens of people and displaced thousands more, and an ongoing tropical storm season. It has borne questions of possible electoral interference including threats of shuttering the federal postal service, intelligence reports of foreign interference and claims from the sitting US President that if he does not win re-election, it is most certainly due to voter fraud. In a year like this, it is utterly unthinkable that a first-term president would have a snowball's chance in hell at winning re-election.

And yet, poll after poll shows President Donald Trump is either tied with or is only narrowly trailing his challenger, former Vice President Joe Biden. The most recent Pew Research poll has Biden ahead with 53% to Trump's 45%. Top pollsters FiveThirtyEight, however, found that in conducting over 40,000 electoral simulations, their model gave Biden a 77% chance of winning to Trump's 23%.

But winning what? In 2016, Hillary Clinton was ahead of Trump in the national polls by a similarly substantial margin. Most polls predicted her win was imminent by at least 80%. And perhaps the polls were correct – Clinton did beat Trump by almost three million votes. But the popular vote does not determine the electoral outcome in the US. In the electoral college system the US uses to elect its president, each state is given a number of votes based on how many representatives it sends to Congress – the House and the Senate. The number of House Representatives is determined by the state's population whereas the number of Senate seats allotted each state is the same – two – creating a rural skew. To win the presidency, a successful candidate needs to secure 270 of the possible 538 electoral college votes – not the popular vote. Indeed, polling experts say that Biden could win the popular vote by 4.5 million votes and still only have a 75% chance of winning the electoral college.

Despite the odds, obstacles and oddities, Trump's support base remains fervent. The President has famously said he could walk down New York City's 5th Avenue and shoot someone and would not lose a single supporter. What was once hyperbole appears to be inching closer to the truth. Trump's supporters forgive every transgression, deny every accusation, reinterpret every misspoken utterance and blame the "fake news" for every mischaracterization. This ardent and unwavering support that may carry Trump to a second term is based on a mixture of what his supporters consider his accomplishments and his characteristics: his appointment of conservative judges, his hard line on immigration, his attempt to dismantle the Affordable Care Act, his lack of adherence to norms of political correctness, his pro-life stance, his unwillingness to take down or rename memorials honoring former confederate war heroes, his defence of civilian militia and known white supremacists, to name just a few.

But the most oft-cited reason, according to numerous polls, for the stark support Trump enjoys is the economy. This seems counterintuitive as the US and much of the world stares down a pandemic-induced recession – restaurants have shuttered, airlines are struggling to stay aloft, parents are juggling work, school and childcare. Regardless, this perception, and whether Biden can shake it or not in the next 40 days, are crucial to the outcome in swing states in the midwestern Rust Belt and the southwestern Sun Belt where the election may be decided. Many of these states have struggled this summer with rising coronavirus infection and death rates as well as rising unemployment and financial insecurity.

And still, many Americans, notably many more Republicans, feel that they are ‘better off’ today than they were four years ago, a measuring standard introduced by Ronald Reagan during the 1980 campaign against incumbent Jimmy Carter. The economy is indeed a crucial litmus test for a president’s re-election prospects.

While unemployment rates are still higher than any other time during the post-World War II era, they have fallen since the onset of the pandemic, the stock market is up and there are signs that the GDP is growing rapidly. The unemployment rate according to the latest figures from the US Bureau of Labor stood at 8.4% for the month of August, although economists argue that the true rate may be understated and is higher than 11% when taking various factors into account.

The latest Financial Times-Peterson Foundation survey, conducted between September 9 and 14, found that 35% of likely voters believe they are better off financially than they were before Trump took office, while 31% say they are worse off. Breaking down the figures, it becomes clear that perception is strongly dictated by party affiliation. The poll shows 51% of Democrats believe they are worse off since Trump became president compared to 7% of Republicans. A recent Gallop poll indicated a 51-percentage-point gap in Republicans’ and Democrats’ ratings of the current economy as excellent or good, and a staggering 66-point partisan gap in the percentages of those who find the economy is improving. The extreme polarization means that party identity plays a more defining roll in voters’ economic evaluations than quantitative measures.

This polarization has led voters to see two completely different realties. President Trump, through reality television and personal marketing, has built an enduring brand with conservative voters, in particular, who point to his successful business ventures and deal-making skills as proof that he is the right person to guide the US economy through the pandemic.

Trump’s supporters accept the gradual erosion of America’s democracy in exchange for what they believe he can do for the economy. Trump’s opponents, however, see a president who bends the institutions of government at his will to his own personal benefit; who rewards loyalty and obedience over competence as seen in the Justice Department, the intelligence community and in the Center for Disease Control; who prioritizes the economy over 200,000 American lives and counting. He recently even went as far as to refuse to commit to a peaceful transfer of power.

Just a few days ago, America lost Ruth Bader Ginsburg, an icon and trailblazing Supreme Court Justice, whose dying wish was to not be replaced before a new president was in office. Trump and Senate Majority leader Mitch McConnell, in defiance of Ginsburg’s wish, vowed that Trump’s nominee will get a vote on the Senate floor before November 3. With the Supreme Court tilting even further to the right, the likelihood that a contested election will be decided in Trump’s favor becomes even more plausible.

America is at a crossroads: Trump supporters may have their say – and their way – on the economy, but they are sacrificing their democracy in the process.

Jiffer Bourguignon, ZBW — Leibniz Information Centre for Economics, Hamburg, Germany.

